# Supplementary material for: Neurofilament Light Chain Levels in Serum and Cerebrospinal Fluid Do Not Correlate with Survival Times in Patients with Prion Disease
Source: Biomolecules. 2024 Dec 25;15(1):8. doi: 10.3390/biom15010008 (PMC11760854; doi:10.3390/biom15010008)
Supplement: Supplementary file 1 [file biomolecules-15-00008-s001.zip › biomolecules-3295531-supplementary.pdf]

biomolecules

Supplementary Materials

Neurofilament Light Chain Levels in Serum and Cerebrospinal Fluid Do Not Correlate with Survival Times in Patients with Prion Disease

Mika Shimamura [1], Kong Weijie [2], Toshiaki Nonaka [3], Koki Kosami [4], Ryusuke Ae [4], Koji Fujita [5], Taiki Matsubayashi [6], Tadashi Tsukamoto [7], Nobuo Sanjo [8,9] and Katsuya Satoh [2,10,*]

1 Biomedical Research Support Center, Nagasaki University, 1-12-4 Sakamoto, Nagasaki 852-8523, Japan; shima-m@nagasaki-u.ac.jp
2 Unit of Medical and Dental Sciences, Department of Health Sciences, Nagasaki University Graduate School of Biomedical Sciences, 1-12-4 Sakamoto, Nagasaki 852-8523, Japan; wj2023nagasaki@gmail.com
3 Division of Cellular and Molecular Biology, Nagasaki University Graduate School of Biomedical Sciences, 1-12-4 Sakamoto, Nagasaki 852-8523, Japan; kbb55417803@ms.nagasaki-u.ac.jp
4 Division of Public Health, Center for Community Medicine, Jichi Medical University, Tochigi 329-0498, Japan; k.kosami@jichi.ac.jp (K.K.); shirouae@jichi.ac.jp (R.A.)
5 Department of Neurology, Tokushima University Graduate School of Biomedical Sciences, 3-18-15 Kuramoto-cho, Tokushima 770-8503, Japan; kfujita@tokushima-u.ac.jp
6 Department of Neurology and Neurological Science, Tokyo Medical and Dental University, Graduate School of Medical and Dental Sciences, 1-5-45 Yushima Bunkyo-ku, Tokyo 113-8510, Japan; taiki.matsubayashi55135@gmail.com
7 Department of Neurology, National Center of Neurology and Psychiatry (NCNP), 4-1-1 Ogawa-Higashi, Kodaira, Tokyo 187-8551, Japan; tukamoto@ncnp.go.jp
8 Department of Internal Medicine, Division of Neurology, Kudanzaka Hospital, 1-6-12 Kudan-minami, Chiyoda-ku, Tokyo 102-0074, Japan; n-sanjo.nuro@tmd.ac.jp
9 Department of Neurology and Neurological Science, Tokyo Medical and Dental University, Graduate School of Medical and Dental Sciences, 2-12-1 Ookayama, Meguro-ku, Tokyo 152-8550, Japan
10 Unit of Medical and Dental Sciences, Department of Health Sciences, Nagasaki University Graduate School of Biomedical Sciences, Nagasaki 852-8523, Japan
* Correspondence: satoh-prion@nagasaki-u.ac.jp

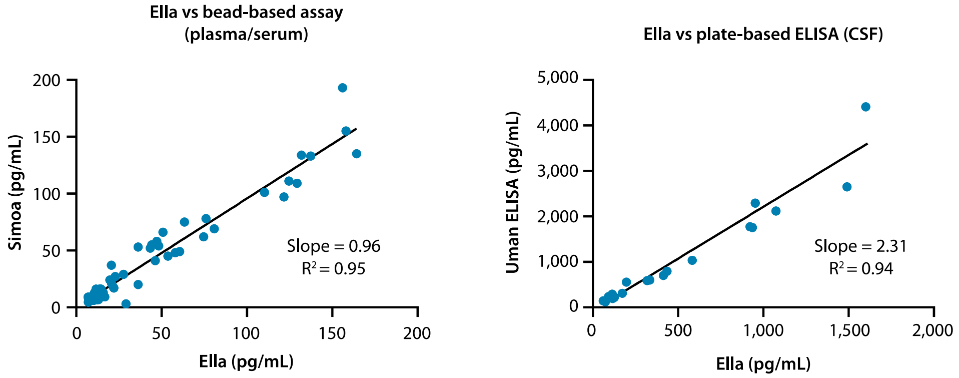

Figure S1. Linear correlation of serum and samples on Ella versus other commercial platforms. NF-L levels measured using the Simple Plex Assay correlated with those quantified by the Simoa Assay and the Uman Diagnostics ELISA. Samples were prepared as per the recommendations of the Re-spective kit manufacturer, and levels of NF-L were measured within the linear range of each assay. The dilution factors were used to back-calculate the original concentration in the samples. These values were then plotted against each other, and a linear curve fitting algorithm was applied.
